# Silent Stroke in Adult Cardiac Surgery: Mechanisms, Clinical Impact, and Preventive Strategies

**DOI:** 10.3390/medicina62040675

**Published:** 2026-04-01

**Authors:** Ignazio Condello, Michele Dell’Aquila, Salvatore Condello, Giorgia Falco, Antonio Totaro, Youssef El Dsouki, Sotirios Prapas, Konstantinos Katsavrias, Augusto D’Onofrio, Joshua Newman, Nirav Patel, Robert Kalimi, Mario Gaudino, Antonio Maria Calafiore

**Affiliations:** 1 School of Medicine and Surgery, University of Insubria, 21100 Varese, Italy; 2 Northwell Health, Cardiovascular Institute, New York, NY 11030, USA; 3 Neuromotor Rehabilitation Unit, Istituti Clinici Scientifici, Maugeri SPA, 92016 Ribera, Italy; 4 Cardiology Unit, University Hospital Consortium Polyclinic of Bari, 70124 Bari, Italy; 5 Department of Medicine and Health Sciences “V. Tiberio”, University of Molise, 86100 Campobasso, Italy; 6 Faculty of Health, Medicine and Life Sciences, Cardiovascular Research Institute Maastricht, 6229 ER Maastricht, The Netherlands; youssefdsouki@hotmail.fr; 7 1st Division of Cardiac Surgery, Henry Dunant Hospital, 115 26 Athens, Greeceam.calafiore@gmail.com (A.M.C.); 8 Division of Cardiac Surgery, University of Rome “Tor Vergata”, 00133 Rome, Italy; 9 Department of Cardiothoracic Surgery, Weill Cornell Medicine, New York, NY 10021, USA

**Keywords:** silent stroke, silent brain injury, cardiac surgery, cardiopulmonary bypass, diffusion-weighted magnetic resonance imaging, cerebral microembolization, cerebral hypoperfusion, oxygen delivery, neuroprotection, postoperative cognitive dysfunction

## Abstract

*Background and Objectives*: Overt perioperative stroke remains a feared complication of adult cardiac surgery. Diffusion-weighted magnetic resonance imaging (DWI-MRI) has revealed a more prevalent form of cerebral injury, termed silent stroke or silent brain injury (SBI). Covert ischemic lesions occur without focal neurological deficits but are increasingly associated with postoperative delirium, cognitive decline, and elevated long-term cerebrovascular risk. Despite growing recognition, the true burden, mechanisms, and clinical relevance of SBI remain incompletely integrated into perioperative practice. *Materials and Methods*: We performed a narrative review of the literature published between January 2000 and December 2025, identified through PubMed/MEDLINE and Scopus. Eligible studies included prospective and retrospective cohorts, randomized trials, systematic reviews, and meta-analyses involving adult patients undergoing coronary artery bypass grafting, valve surgery, or minimally invasive cardiac procedures, with or without cardiopulmonary bypass, and reporting MRI-detected ischemic lesions or validated surrogate markers of cerebral injury. Pediatric studies, transcatheter interventions, case reports, and non-English publications were excluded. Sixty studies met the inclusion criteria. *Results*: Silent stroke occurred more frequently than clinically apparent stroke, with new DWI-MRI lesions detected in approximately 20–60% of patients following cardiac surgery. Lesions were typically small, multifocal, and embolic in distribution, predominantly affecting cortical and watershed regions. Cardiopulmonary bypass-related factors, including aortic manipulation, cerebral microembolization, hemodilution, hypoperfusion, and impaired oxygen delivery, emerged as key contributors. Several studies demonstrated associations between SBI burden and postoperative delirium, early cognitive dysfunction, and functional decline. Perfusion-based neuroprotective strategies showed mechanistic benefit, although no single intervention conclusively prevented SBI. *Conclusions*: Silent stroke represents the most frequent form of neurological injury in adult cardiac surgery. Evidence suggests that these covert lesions reflect clinically meaningful cerebral injury, with potential short- and long-term consequences. Recognition of silent stroke as a relevant neurological endpoint supports a shift toward multimodal, perfusion-driven neuroprotective strategies and the routine incorporation of MRI-based outcomes in future cardiac surgical research.

## 1. Introduction

Neurological injury remains one of the most consequential complications of adult cardiac surgery. Advances in diffusion-weighted magnetic resonance imaging (DWI-MRI) have fundamentally changed the understanding of perioperative cerebral injury, revealing a high burden of previously unrecognized ischemic lesions [1,2,3,4,5,6,7,8]. These MRI-detected infarcts, commonly referred to as silent stroke or silent brain injury (SBI), occur in the absence of focal neurological deficits yet reflect objective structural cerebral injury. Mechanistic investigations have demonstrated that cerebral injury during cardiac surgery arises from a complex interaction between embolic phenomena and perfusion-related factors. Randomized and observational studies have shown that perfusion pressure targets alone do not fully prevent cerebral injury [9], while transcranial Doppler (TCD) investigations have consistently documented substantial microembolic load during cardiopulmonary bypass (CPB) and aortic manipulation [10,11]. In parallel, reduced hematocrit and impaired oxygen delivery during CPB have been associated with increased neurological risk [12,13], and regional cerebral oxygen desaturation has correlated with MRI-detected ischemic lesions [14]. Although the incidence of clinically overt perioperative stroke has declined to approximately 1–3% in contemporary practice [15], MRI studies have demonstrated that new ischemic lesions occur in a substantially higher proportion of patients. Importantly, these lesions are not necessarily benign. Early investigations demonstrated an association between DWI-detected lesions and postoperative neurocognitive decline [16]. Editorial perspectives have emphasized that the distinction between “overt” and “covert” stroke often reflects limitations in detection rather than absence of injury [17,18] Comprehensive reviews have further highlighted the clinical relevance of silent brain infarction and its relationship to postoperative cognitive dysfunction and long-term neurological vulnerability [19,20]. More recent prospective MRI studies have confirmed that new ischemic lesions occur in approximately 20–60% of patients undergoing coronary artery bypass grafting, valve surgery, or minimally invasive cardiac procedures [21,22]. These lesions are typically small, multifocal, and embolic in distribution, frequently involving cortical and watershed territories. Increasing evidence also links silent cerebral infarction to postoperative delirium and cognitive impairment across invasive cardiovascular procedures [23].

Beyond early postoperative outcomes, subclinical brain injury has been associated with increased long-term risk of symptomatic stroke and dementia in broader cardiovascular populations [24]. While perioperative stroke remains a feared complication [25,26], meta-analytic evidence has demonstrated that silent brain infarction occurs several-fold more frequently than clinically apparent stroke after cardiac surgery [27,28,29,30,31]. Despite growing recognition of silent stroke as the most frequent neurological injury associated with adult cardiac surgery, several important gaps remain. MRI-defined lesions are not routinely incorporated into outcome reporting or quality frameworks; mechanistic pathways linking embolic load, oxygen delivery, autoregulation, and gas management remain insufficiently integrated; and heterogeneity in imaging protocols limits comparability across studies. Accordingly, the aim of this narrative review is to synthesize current evidence on silent stroke in adult cardiac surgery, focusing on incidence, imaging characteristics, pathophysiological determinants, perfusion-related mechanisms, and preventive strategies.

Unlike prior reviews focusing primarily on imaging incidence or postoperative cognitive dysfunction, this review integrates MRI findings with perfusion physiology, gas management strategies, autoregulatory considerations, and emerging endothelial mechanisms, providing a mechanistically unified framework for perioperative neuroprotection.

## 2. Methods

**Figure 1:**
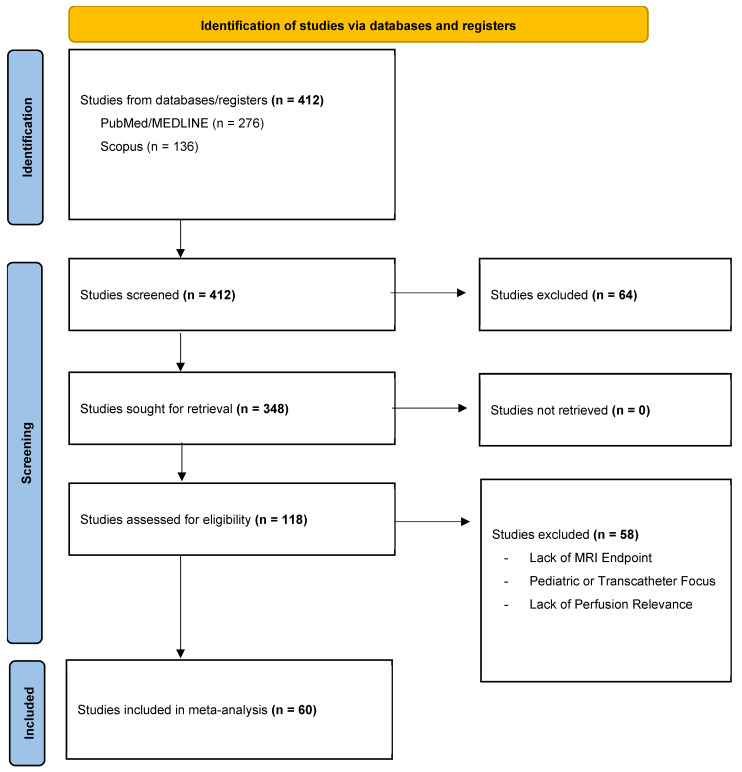
PRISMA flow diagram of literature search and study selection process.

This article was developed as a narrative review aimed at providing an integrated overview of silent stroke and SBI in adult cardiac surgery. Given the heterogeneity of study designs, imaging protocols, surgical techniques, and perfusion strategies across the available literature, a narrative approach was selected to allow contextual interpretation of evidence and mechanistic integration rather than quantitative aggregation of results. The literature considered for this review was identified through a broad and exploratory search of the PubMed/MEDLINE and Scopus databases. The search focused on publications from January 2000 to December 2025, corresponding to the period in which DWI-MRI became widely available for the detection of perioperative cerebral ischemic injury. Search terms were selected to capture the evolving terminology and concepts related to subclinical cerebral injury in cardiac surgery and included combinations of “silent stroke,” “silent brain infarction,” “silent brain injury,” “covert stroke,” “diffusion-weighted MRI,” “cardiac surgery,” “coronary artery bypass grafting,” “valve surgery,” “cardiopulmonary bypass,” “perfusion,” “microembolization,” “oxygen delivery,” “cerebral oxygenation,” and “neuroprotection.” Reference lists of key articles, reviews, and meta-analyses were also examined to identify additional relevant studies. The literature was not restricted to a single study design. Prospective and retrospective clinical studies, randomized trials, systematic reviews, and meta-analyses were all considered, provided they addressed silent cerebral ischemic injury in adult cardiac surgical populations. Emphasis was placed on studies using postoperative MRI to detect silent cerebral lesions, as well as investigations exploring perfusion-related mechanisms of brain injury, including CPB configuration, aortic manipulation, oxygen delivery, perfusion pressure, hemodilution, and cerebral monitoring. Studies focusing exclusively on pediatric populations, transcatheter interventions, experimental models, or isolated clinical stroke without imaging correlation were not considered central to the scope of this review. Studies were selected based on their relevance to the core themes of the review: incidence and imaging characteristics of silent stroke, underlying pathophysiological mechanisms, the contribution of perfusion strategies to cerebral injury, clinical and cognitive implications, and preventive or neuroprotective approaches. Data extraction was qualitative and focused on conceptual content, mechanistic insight, and consistency of findings across studies. Given the substantial variability in imaging timing, neurological assessment, perfusion protocols, and outcome definitions, no formal quantitative synthesis was attempted. Instead, the evidence was narratively integrated and organized into thematic sections to highlight converging patterns, unresolved controversies, and knowledge gaps. Although this study was designed as a narrative review and no formal quantitative synthesis was performed, study identification and selection were documented using a PRISMA-style flow framework to enhance transparency (Figure 1). The initial database search yielded 412 records. After removal of duplicates (n = 64), 348 titles and abstracts were screened. Of these, 118 full-text articles were assessed for eligibility. Fifty-eight articles were excluded due to lack of MRI-defined endpoints, pediatric or transcatheter focus, or insufficient relevance to perfusion-related mechanisms. Ultimately, 60 publications met the inclusion criteria and were narratively synthesized.

## 3. Results

The literature search identified a broad and heterogeneous body of evidence addressing silent stroke and SBI in adult cardiac surgery. A total of 60 publications were considered relevant and were included in this narrative synthesis [1,2,3,4,5,6,7,8,9,10,11,12,13,14,15,16,17,18,19,20,21,22,23,24,25,26,27,28,29,30,31,32,33,34,35,36,37,38,39,40,41,42,43,44,45,46,47,48,49,50,51,52,53]. The retrieved literature comprised prospective and retrospective observational studies, randomized controlled trials, systematic reviews, meta-analyses, mechanistic perfusion investigations, and editorial perspectives. Collectively, these studies encompassed a wide range of cardiac surgical procedures, including CABG, valve surgery, and minimally invasive cardiac operations, performed with or without CPB. Neurological outcomes were most frequently assessed using DWI-MRI, which represents the reference standard for the detection of silent cerebral ischemic lesions. Several studies complemented MRI findings with TCD monitoring of cerebral microemboli [10,11,34], near-infrared spectroscopy (NIRS)-based assessment of regional cerebral oxygen saturation (rSO_2_) [14,20,21,22], formal neuropsychological testing [6,7,16,43], or biochemical markers of neuronal injury [13]. Given the substantial heterogeneity in study design, imaging protocols, timing of assessments, and perfusion strategies, findings were synthesized qualitatively and organized into thematic domains.

### 3.1. Incidence of Silent Stroke After Cardiac Surgery

Across the literature, silent stroke consistently emerged as the most frequent neurological complication following adult cardiac surgery [1,4,5,6,7]. These findings were reproducible across different institutions, imaging protocols, and patient populations [3,43,45]. Meta-analytic evidence further underscored the magnitude of this phenomenon. Guo et al. reported a pooled incidence of silent brain infarction exceeding 40% after on-pump cardiac surgery, with significant heterogeneity driven by differences in CPB duration, surgical complexity, and patient-related risk factors [8,29]. Systematic reviews comparing radiographic and clinical outcomes consistently demonstrated that silent infarcts occur several-fold more frequently than clinically apparent stroke, confirming that overt neurological events substantially underestimate the true burden of perioperative cerebral injury [30,31]. Procedure type influenced but did not eliminate risk. Silent brain lesions were reported following on-pump CABG, off-pump CABG, valve replacement, minimally invasive cardiac surgery, and combined procedures [1,2,4,6,46,47]. Although off-pump and anaortic techniques were associated with lower lesion rates compared with conventional on-pump surgery, silent infarcts remained detectable even in these settings [2,41,47].

### 3.2. Imaging Characteristics and Lesion Distribution

Silent strokes detected after cardiac surgery were typically small, multifocal, and embolic in appearance. DWI-MRI studies consistently described punctate ischemic lesions distributed across multiple vascular territories, with preferential involvement of cortical and watershed regions [1,4,5,45]. These lesion patterns strongly suggested embolic or embolic–hypoperfusion mechanisms rather than large-vessel occlusion. Advanced imaging techniques further refined lesion characterization. Susceptibility-weighted imaging revealed microhemorrhagic components or embolic signatures accompanying ischemic lesions, reinforcing the role of microembolization [3]. Lesion burden varied widely between patients, ranging from single punctate infarcts to multiple bilateral lesions. Several studies suggested that higher lesion number and volume were associated with worse neurocognitive outcomes [6,7,16,47]. Differences in MRI protocols and postoperative imaging timing contributed to variability in reported incidence and lesion burden across studies [30,39].

### 3.3. Cerebral Microembolization

Cerebral microembolization emerged as the dominant mechanism underlying silent stroke in cardiac surgery. TCD studies consistently demonstrated high embolic loads during CPB, particularly during aortic cannulation, cross-clamping, side-biting, and de-airing maneuvers [10,11]. Both gaseous and particulate emboli were detected, with embolic counts generally higher during CPB and extensive aortic manipulation [11,34]. Off-pump CABG reduced but did not abolish embolization. In patients with severe aortic atheroma, off-pump and “no-touch” strategies significantly reduced the incidence of MRI-detected ischemic lesions compared with conventional on-pump surgery [2]. However, embolic signals persisted even during off-pump procedures, reflecting ongoing sources of gaseous and particulate emboli related to cardiac manipulation and surgical instrumentation [11].

### 3.4. Cerebral Hypoperfusion and Oxygen Delivery

In addition to embolization, impaired cerebral perfusion and oxygen delivery contributed to SBI. A randomized controlled trial demonstrated that targeting higher mean arterial pressure (MAP) during CPB did not reduce the incidence or volume of MRI-detected cerebral lesions, indicating that perfusion pressure alone is insufficient for cerebral protection [9]. Conversely, hemodilution and reduced oxygen delivery emerged as important contributors. Low nadir hematocrit during CPB was independently associated with increased neurological risk [12]. Studies correlating indexed oxygen delivery with neuron-specific injury biomarkers demonstrated biochemical evidence of cerebral injury even in the absence of clinical stroke [13]. Cerebral oxygen desaturation detected by NIRS was also associated with postoperative ischemic lesions on MRI, supporting a role for metabolic insufficiency and supply–demand mismatch in SBI pathogenesis [14].

### 3.5. Hyperoxia, Cerebral Vasoconstriction, and Microcirculatory Effects

The role of hyperoxia as a modifier of cerebral injury during cardiac surgery remains controversial. While high arterial oxygen tension is routinely employed during CPB to maximize systemic oxygen delivery, physiological and experimental data indicate that hyperoxia may induce cerebral vasoconstriction, potentially reducing cerebral blood flow (CBF) and impairing microcirculatory oxygen delivery. Cerebral autoregulation is sensitive to arterial oxygen tension, and hyperoxia has been shown to reduce CBF despite preserved or increased arterial oxygen content. This raises concern that excessive oxygen tension during CPB may exacerbate hypoperfusion in vulnerable regions, such as watershed zones, which are commonly affected by silent ischemic lesions [24,26]. Clinical studies evaluating cerebral oxygenation using NIRS have demonstrated that increases in arterial oxygen tension do not necessarily translate into proportional improvements in rSO_2_ and may, in some cases, be associated with unchanged or reduced values, consistent with vasoconstrictive effects [14,20,21,22]. These observations suggest a potential dissociation between arterial oxygen content and effective tissue oxygen delivery under hyperoxic conditions. Conversely, other investigations failed to demonstrate a clear association between hyperoxia and adverse neurological outcomes. In many clinical studies, hyperoxia was confounded by simultaneous changes in pump flow, perfusion pressure, hematocrit, temperature, and carbon dioxide management, limiting causal inference. Large observational and interventional studies of perfusion strategies did not consistently identify hyperoxia as an independent predictor of silent stroke or postoperative cognitive dysfunction, and MRI-based evidence directly linking oxygen tension targets to silent brain infarction remains sparse [9,12,13,39]. Overall, the literature provides conflicting evidence regarding the impact of hyperoxia on SBI. While mechanistic considerations suggest a plausible contribution through cerebral vasoconstriction and microcirculatory dysfunction, definitive clinical data are lacking. No randomized trials have specifically evaluated oxygen tension targets using MRI-defined silent stroke as a primary endpoint, highlighting an important gap in perfusion-focused neuroprotection research.

### 3.6. Perfusion Strategies and Circuit Configuration

Perfusion-related variables significantly influenced cerebral vulnerability. Miniaturized extracorporeal circulation systems were developed to reduce hemodilution, inflammatory activation, and air–blood interface exposure. Observational studies and meta-analyses suggested improved physiological profiles and reduced clinical neurological complications with these systems; however, consistent reductions in MRI-detected silent stroke have not yet been demonstrated [17,18]. Arterial line filtration reduced TCD-detected embolic counts but did not reliably translate into lower rates of silent cerebral infarction on MRI [11]. Experimental work demonstrated that hypobaric oxygenation eliminated the majority of gaseous microemboli within CPB circuits, representing a promising mechanistic advance awaiting clinical validation [35]. Carbon dioxide field flooding during open-chamber procedures was associated with reduced postoperative neurological impairment, supporting the role of gaseous emboli in SBI [19]. Carbon dioxide management (alpha-stat versus pH-stat) influences cerebral autoregulation, microcirculatory flow distribution, and embolic washout during hypothermic CPB, representing an additional modifiable determinant of SBI susceptibility [20,21,22].

### 3.7. Cannulation Strategy and Surgical Technique

Arterial cannulation strategy influenced embolic risk, particularly in minimally invasive cardiac surgery. Retrograde femoral arterial perfusion raised concerns regarding embolic potential in patients with aortic or peripheral vascular disease [25], whereas axillary artery cannulation restored antegrade cerebral flow and was associated with favorable neurological outcomes in selected cohorts [23]. MRI-based studies demonstrated that silent brain infarction remained detectable after minimally invasive procedures, even with careful patient selection and optimized perfusion strategies [46,51]. Aortic occlusion technique represented another contributor to embolic risk. Comparative studies of external cross-clamping and endoaortic balloon occlusion reported similar rates of clinical stroke but raised concerns regarding microembolization and silent injury, which remain insufficiently characterized by systematic MRI evaluation [32,33].

### 3.8. Clinical and Cognitive Consequences

Although silent strokes lack overt neurological symptoms, multiple studies demonstrated clinically relevant consequences. Early neuropsychological investigations identified postoperative cognitive dysfunction in patients with MRI-detected ischemic lesions [6,7,43]. Direct associations between lesion burden and impairment in executive function, memory, and attention were reported [7,16]. More recent studies linked silent brain infarction to postoperative delirium, early functional decline, and impaired activities of daily living [47,48,53]. High-risk populations, including elderly patients and those with pre-existing silent infarction or cerebrovascular disease, exhibited higher rates of postoperative neurological complications [44,54]. Longitudinal data from broader cardiovascular literature further suggested increased long-term risk of symptomatic stroke and dementia in patients with silent cerebral infarcts [24,30,31,40,50]. Editorial commentaries emphasized that the distinction between “silent” and “overt” stroke often reflects limitations in detection rather than absence of injury. Vekstein and Lin, Worku and Gaudino, and Rajagopal argued that MRI-based detection has redefined neurological outcomes in cardiac surgery and that silent lesions should no longer be dismissed as incidental findings [36,37,38] (Table 1).

## 4. Discussion

Across available evidence, SBI in adult cardiac surgery reflects the interaction between embolic burden and impaired cerebral oxygen delivery within a context of variable autoregulatory reserve. [1,2,3,4,5,6,7,8,29,30,31,39,45,46,47]. In contrast, reported rates of clinical stroke vary widely and remain comparatively low, reinforcing the notion that traditional neurological endpoints underestimate the true burden of cerebral injury. Comparisons between on-pump and off-pump techniques suggest a relative reduction in silent brain infarcts with off-pump or anaortic strategies, particularly in patients with advanced aortic atherosclerosis [2,41,47,54]. However, silent lesions persist even in off-pump cohorts, indicating that embolization from non-aortic sources and perfusion-related factors continue to play a role [11,34]. Thus, while surgical technique modifies risk, it does not fully mitigate cerebral injury. Similarly, perfusion-focused comparisons highlight important distinctions between pressure-based and delivery-based strategies. Randomized data indicate that higher MAP targets during CPB do not reduce MRI-detected silent stroke [9], whereas observational and biomarker-based studies implicate low hematocrit and impaired oxygen delivery as more relevant determinants of cerebral injury [12,13]. These findings suggest that comparisons based solely on perfusion pressure are insufficient and that oxygen delivery-centered paradigms may better capture cerebral risk. The literature addressing hyperoxia illustrates the complexity of comparative interpretation. Physiological and monitoring studies suggest that hyperoxia may induce cerebral vasoconstriction and reduce effective microcirculatory flow [14,20,21,22,24,26], whereas clinical outcome studies have not consistently demonstrated a direct association with silent stroke or cognitive decline [9,12,13,39]. This discrepancy underscores the difficulty of isolating individual perfusion variables in a highly controlled and interdependent physiological environment. Overall, comparative analysis across studies supports a multifactorial model of SBI, in which embolic load, cerebral perfusion, oxygen delivery, and microvascular regulation interact dynamically rather than acting as isolated determinants.

### 4.1. Limitations of the Available Evidence

**Figure 2:**
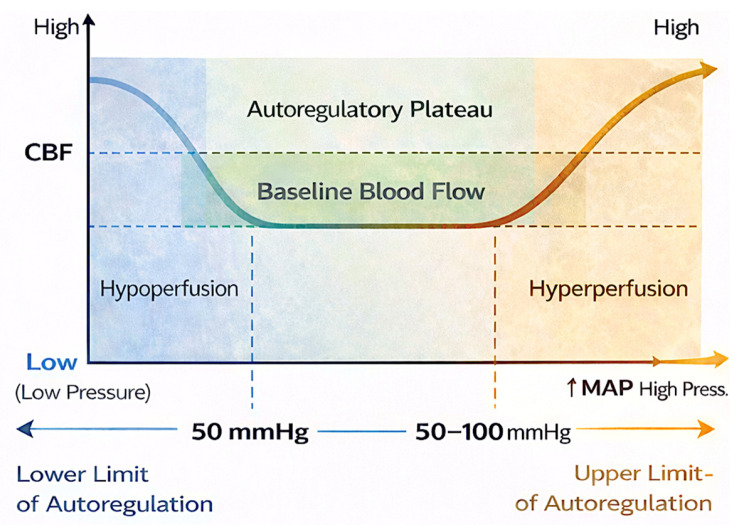
Cerebral autoregulation and mean arterial pressure (MAP). Under physiological conditions, cerebral autoregulation maintains relatively constant cerebral blood flow (CBF) across a range of MAP values. Within this autoregulatory plateau, cerebrovascular resistance adjusts to buffer fluctuations in perfusion pressure. When MAP falls below the lower limit of autoregulation or exceeds the upper limit, autoregulatory capacity is lost and cerebral blood flow becomes pressure-passive, meaning that CBF varies directly with systemic blood pressure. In this state, reductions in MAP may lead to cerebral hypoperfusion and ischemia, whereas excessive pressure may increase the risk of hyperperfusion injury.

Cerebral autoregulation plays a central role in maintaining stable CBF across a wide range of perfusion pressures and metabolic conditions. During cardiac surgery, and particularly during CPB, autoregulatory mechanisms may be impaired by advanced age, cerebrovascular disease, systemic inflammation, hemodilution, anesthetic agents, and non-physiological flow patterns (Figure 2). Disruption of autoregulation increases cerebral vulnerability to both hypoperfusion and embolic injury, thereby facilitating the development of silent brain infarction. Emerging evidence suggests that autoregulatory failure may be particularly relevant in patients who develop silent ischemic lesions, as these injuries frequently localize to watershed regions that are highly sensitive to pressure- and flow-dependent changes. In this context, global perfusion targets based on fixed MAP thresholds may be insufficient to ensure adequate cerebral perfusion at the individual patient level. Instead, CBF may become pressure-passive, rendering the brain susceptible to ischemia during even modest reductions in perfusion pressure or oxygen delivery. Despite its importance, routine intraoperative monitoring of cerebral autoregulation remains limited. NIRS, while widely used, provides only indirect information on regional cerebral oxygenation, and does not directly assess autoregulatory capacity. Changes in rSO_2_ may reflect alterations in arterial oxygen content, venous saturation, or extracranial contamination rather than true CBF. Similarly, TCD allows assessment of flow velocity but is operator-dependent, not universally applicable, and rarely used continuously throughout surgery. Advanced autoregulation monitoring techniques such as correlation-based indices derived from blood pressure and cerebral oxygenation, or flow signals have shown promise in research settings but are not yet standardized or widely implemented in clinical practice [55]. As a result, clinicians currently lack reliable, real-time tools to identify the lower and upper limits of autoregulation during cardiac surgery, limiting the ability to individualize perfusion and gas management strategies. These monitoring limitations represent a critical gap in current neuroprotective approaches. Future integration of autoregulation-guided perfusion targets, combined with multimodal neuromonitoring and imaging-based outcome validation, may allow more precise identification of patients at risk for SBI and support individualized strategies to preserve cerebral perfusion and minimize neurological injury [56]. Several important limitations constrain the interpretation of the existing literature. First, substantial heterogeneity exists in MRI protocols, including timing of postoperative imaging, magnetic field strength, diffusion parameters, and lesion definitions. Because diffusion-restricted lesions can evolve or resolve over time, differences in imaging windows likely contribute to the wide variability in reported incidence [30,39,57]. Second, many studies are underpowered to detect associations between silent stroke and long-term clinical outcomes. While associations with postoperative delirium and early cognitive dysfunction are relatively consistent [6,7,16,43,47,48,53,58], long-term cognitive and functional trajectories remain insufficiently characterized. Few studies include extended follow-up or standardized neurocognitive batteries, limiting causal inference. Third, confounding is pervasive, particularly in studies examining perfusion variables and oxygen management. Hyperoxia, perfusion pressure, pump flow, hematocrit, temperature, and carbon dioxide tension are often modified simultaneously, making it difficult to isolate independent effects [9,12,13,14,20,21,22]. As a result, many conclusions regarding perfusion-related risk factors remain associative rather than causal. Fourth, the majority of available data are derived from single-center observational studies or secondary analyses. Randomized controlled trials with MRI-defined silent stroke as a prespecified endpoint are rare, and when present, are often not powered for neurological outcomes [9,59]. Additionally, many potentially relevant studies rely on surrogate markers of cerebral injury rather than direct imaging confirmation. Finally, silent stroke itself remains a heterogeneous construct. Lesion number, volume, location, and cumulative burden are rarely standardized, and thresholds for clinically meaningful injury are not well defined. This heterogeneity complicates comparisons across studies and limits translation into routine clinical decision-making [36,37,38]. Another potential confounding factor relates to the influence of anesthetic agents on cerebrovascular physiology. Volatile anesthetics and intravenous agents may differentially affect cerebral metabolic rate, vascular tone, and the cerebrovascular response to PaCO_2_. Variability in anesthetic protocols across studies may therefore influence PaCO_2_-mediated vasoreactivity and CBF regulation during CPB, potentially modifying the relationship between gas management strategies and the risk of silent cerebral injury [35,36,37,38,39,40,41,42,43,44,45,46,47,48,49,50,51,52,53,54,55,56,57,58,59,60].

Finally, this work was conducted as a narrative review and does not constitute a systematic review or meta-analysis. No formal risk-of-bias assessment, quantitative pooling of data, or predefined protocol registration was performed. The objective was integrative and mechanistic synthesis rather than statistical aggregation. Consequently, conclusions should be interpreted as conceptually integrative rather than quantitatively definitive.

### 4.2. Future Perspectives and Research Directions

The findings of this review highlight several priorities for future research and clinical refinement aimed at reducing SBI in adult cardiac surgery. First, the adoption of standardized MRI protocols including timing of postoperative imaging, acquisition parameters, and quantitative reporting of lesion number, volume, and distribution is essential to improve comparability across studies and enable meaningful meta-analyses. Similarly, the development of consensus definitions for silent stroke and SBI would strengthen interpretability and facilitate the integration of imaging-based neurological endpoints into clinical research and quality assessment frameworks [24,39,40]. Second, future randomized and prospective studies should incorporate MRI-detected silent stroke as a primary or secondary endpoint. This is particularly relevant for evaluating perfusion strategies, oxygen tension targets, embolic mitigation technologies, and CPB circuit innovations. Trials focusing on optimization of oxygen delivery—rather than perfusion pressure alone may be especially informative, given accumulating evidence that cerebral injury is more closely related to hemodilution and impaired oxygen delivery than to mean arterial pressure targets [9,12,13]. Third, several technical and procedural strategies aimed at reducing embolic load warrant more systematic evaluation using imaging-based neurological endpoints. Routine use of epiaortic ultrasound scanning to guide cannulation and cross-clamp placement represents a critical yet underutilized intervention. Accurate identification of aortic atheroma burden allows avoidance of high-risk segments and supports individualized cannulation strategies, particularly in elderly and high-risk patients. Despite strong mechanistic rationale, the impact of epiaortic scanning on silent stroke incidence remains insufficiently characterized by MRI-based studies and should be a priority for future investigation [2,10,11,34]. In parallel, aortic cannulation technique deserves greater attention. Cannula insertion with alignment of flow parallel to the aortic lumen rather than directed toward the aortic wall may reduce shear stress, plaque disruption, and downstream embolization. Although this concept is widely accepted in surgical practice, its neurological impact has not been rigorously evaluated using sensitive imaging endpoints and remains an important area for prospective study. Fourth, standardization of de-airing strategies represents another promising but underexplored avenue for embolic risk reduction. While gaseous microembolization is widely recognized as a contributor to cerebral injury, de-airing techniques remain highly variable across centers and operators. Protocolized de-airing before cross-clamp removal—integrating surgical maneuvers, perfusion strategies, and anesthetic management—may reduce cerebral embolic load. In particular, coordinated and isovolumetric management of pulmonary, intravascular, and ventilatory volumes may help minimize air entrainment and embolic release during cardiac reperfusion. Future studies should evaluate standardized de-airing protocols using TCD and MRI-defined silent stroke as objective outcome measures [11,19,35]. Fifth, the combined role of hyperoxia and hypocapnia as modulators of CBF and microcirculatory perfusion warrants focused investigation. Both conditions are common during CPB and early reperfusion and are potent, synergistic inducers of cerebral vasoconstriction. While hyperoxia increases arterial oxygen content, it may paradoxically reduce CBF through oxygen-induced vasoconstriction; similarly, hypocapnia produces a well-established reduction in CBF through CO_2_-mediated vasoreactivity. When present simultaneously, hyperoxia and hypocapnia may significantly impair effective cerebral oxygen delivery despite apparently adequate systemic perfusion [14,20,21,22,24,26]. Carefully designed trials controlling PaCO_2_ within a normocapnic range while testing defined oxygen tension targets are therefore needed. Sixth, pharmacological modulation of endothelial function and nitric oxide (NO) signaling represents an emerging and largely unexplored avenue for neuroprotection in cardiac surgery. CPB is associated with endothelial dysfunction, impaired NO bioavailability, oxidative stress, and altered microvascular reactivity—factors that may exacerbate cerebral vasoconstriction and microcirculatory impairment. Pharmacologic agents capable of modulating endothelial tone, vascular reactivity, and NO pathways may therefore influence susceptibility to SBI. In this context, the role of vasoactive drugs commonly used during CPB including catecholamines and vasopressors requires further scrutiny, as these agents may differentially affect cerebral microcirculation beyond their effects on systemic blood pressure. Likewise, strategies aimed at preserving or restoring endothelial NO availability may offer neuroprotective potential. Substrates and mediators of NO synthesis, such as L-arginine, represent biologically plausible interventions capable of enhancing endothelial-dependent vasodilation and counteracting hyperoxia- or hypocapnia-induced vasoconstriction. Although current evidence remains preliminary and largely indirect, modulation of the NO pathway may help maintain cerebral microvascular flow during periods of heightened ischemic vulnerability. Future studies should therefore explore whether targeted endothelial support through pharmacological agents, metabolic substrates, or NO-modulating strategies can favorably influence cerebral perfusion and reduce MRI-detected SBI. Importantly, such interventions should be evaluated using robust neuroimaging and neurocognitive endpoints rather than solely systemic hemodynamic measures. Finally, greater integration of neuroimaging with longitudinal neurocognitive and functional outcomes is essential. Silent stroke should be evaluated not merely as an imaging finding but as a marker of long-term neurological vulnerability, particularly in elderly patients and those with pre-existing cerebrovascular disease [30,31,40,44,49,54,55,56,57,58]. Multicenter studies combining standardized MRI, cognitive assessment, and functional follow-up will be crucial to defining clinically meaningful thresholds of SBI. From a clinical standpoint, silent stroke should be recognized as a sensitive indicator of cerebral injury and incorporated into both research and quality improvement frameworks. Rather than seeking a single protective intervention, future strategies should emphasize multimodal, individualized neuroprotection, integrating embolic reduction (through aortic scanning, optimized cannulation, and standardized de-airing), optimized oxygen delivery, tailored perfusion targets, avoidance of hypocapnia, judicious oxygen management, and support of endothelial function through pharmacological and metabolic pathways. Importantly, the implementation of a perfusion-driven neuroprotective strategy should not be considered dependent on advanced autoregulation monitoring technologies alone. In resource-limited environments where continuous autoregulatory assessment is not available, pragmatic approaches based on physiological surrogates may still support cerebral protection. These include maintenance of adequate indexed oxygen delivery during CPB, avoidance of excessive hemodilution, prevention of prolonged cerebral oxygen desaturation detected by NIRS when available, and maintenance of normocapnia to preserve CO_2_-mediated cerebrovascular reactivity. In such contexts, simplified perfusion protocols emphasizing stable pump flow, adequate hematocrit, and careful gas management may approximate key elements of a perfusion-driven strategy while remaining feasible across diverse clinical settings.

## 5. Conclusions

Silent stroke is the most frequent neurological injury in adult cardiac surgery and occurs substantially more often than clinically overt stroke. MRI-detected lesions are consistently associated with postoperative delirium, cognitive dysfunction, and long-term cerebrovascular vulnerability. Current evidence supports a multifactorial pathogenesis involving microembolization, impaired oxygen delivery, dysregulated autoregulation, and gas management variables. No single intervention eliminates risk. Future research should prioritize standardized MRI-based endpoints, autoregulation-guided perfusion strategies, optimization of oxygen delivery rather than pressure alone, embolic mitigation techniques, and endothelial-targeted interventions. Recognition of silent stroke as a clinically meaningful endpoint provides a framework for advancing individualized neuroprotection in cardiac surgery.

## Figures and Tables

**Table 1:** Summary of Key Findings on Silent Stroke in Adult Cardiac Surgery.

Thematic Domain	Main Findings	Key Evidence/Methods	Representative References
Incidence of Silent Stroke	Silent cerebral ischemic lesions occur in 20–60% of patients after adult cardiac surgery, markedly exceeding the incidence of clinically overt stroke. Risk is reduced but not abolished with off-pump or anaortic techniques.	DWI-MRI-based observational studies; prospective cohorts; meta-analyses	[1,2,3,4,5,6,7,8,29,30,31,41,45,46,47]
Imaging Characteristics	Lesions are typically small, multifocal, punctate, and distributed across multiple vascular territories, with preferential involvement of cortical and watershed regions. Lesion burden varies widely among patients.	DWI-MRI; susceptibility-weighted imaging (SWI)	[1,3,4,5,6,7,16,30,39,45,47]
Cerebral Microembolization	Microembolization is the dominant mechanism of silent stroke. Embolic load is highest during aortic manipulation (cannulation, cross-clamping, side-biting, de-airing). Both gaseous and particulate emboli contribute.	TCD; MRI lesion patterns	[2,10,11,34]
Cerebral Hypoperfusion and Oxygen Delivery	Perfusion pressure alone does not protect against silent stroke. Hemodilution and reduced oxygen delivery are associated with biochemical and imaging markers of cerebral injury.	Randomized trials; NIRS monitoring; biomarker studies	[9,12,13,14]
Hyperoxia and Cerebral Vasoconstriction	Hyperoxia may induce cerebral vasoconstriction and impair microcirculatory oxygen delivery, particularly in watershed regions. Clinical evidence is conflicting, with no definitive MRI-based trials targeting oxygen tension.	Physiological studies; NIRS; observational clinical studies	[9,12,13,14,20,21,22,24,26,39]
Perfusion Strategies and CPB Configuration	Miniaturized circuits, embolic filtration, and hypobaric oxygenation reduce embolic load and inflammatory activation, but consistent reductions in MRI-detected silent stroke have not been demonstrated.	Observational studies; meta-analyses; experimental models	[17,18,19,35]
Cannulation Strategy and Surgical Technique	Retrograde femoral perfusion may increase embolic risk in selected patients, while axillary cannulation restores antegrade flow. Silent infarcts remain detectable even with optimized minimally invasive strategies.	MRI-based cohort studies; comparative surgical analyses	[23,25,32,33,46,51]
Clinical and Cognitive Consequences	Silent stroke is associated with postoperative delirium, cognitive dysfunction, and functional decline. Higher lesion burden correlates with worse neurocognitive outcomes.	Neuropsychological testing; longitudinal observational studies	[6,7,16,43,47,48,53,54]
Long-Term Neurological Impact	Silent cerebral infarcts are associated with increased long-term risk of symptomatic stroke, dementia, and mortality.	Longitudinal studies; systematic reviews	[24,30,31,40,49,50]
Conceptual and Editorial Perspectives	MRI has redefined neurological outcomes in cardiac surgery. Silent stroke should be considered a clinically meaningful endpoint rather than an incidental finding.	Editorial commentaries; narrative reviews	[36,37,38,39]

## Data Availability

No new data were created or analyzed in this study. Data sharing is not applicable to this article.

## Abbreviations

The following abbreviations are used in this manuscript:

CABG	Coronary Artery Bypass Grafting
CBF	Cerebral Blood Flow
CPB	Cardiopulmonary Bypass
DWI	Diffusion-Weighted Imaging
DWI-MRI	Diffusion-Weighted Magnetic Resonance Imaging
MAP	Mean Arterial Pressure
MRI	Magnetic Resonance Imaging
NIRS	Near-Infrared Spectroscopy
NO	Nitric Oxide
PaCO_2_	Partial Pressure of Arterial Carbon Dioxide
PaO_2_	Partial Pressure of Arterial Oxygen
rSO_2_	Regional Cerebral Oxygen Saturation
SBI	Silent Brain Injury
SWI	Susceptibility-Weighted Imaging
TCD	Transcranial Doppler
